# Sex differences in the association between amygdala volumes and non‐emotional memory retrieval performance

**DOI:** 10.1002/alz.091371

**Published:** 2025-01-09

**Authors:** Darlingtina Esiaka, Emily Willis, Lucas Broster, David K Powell, Gregory A Jicha, Yang Jiang

**Affiliations:** ^1^ University of Kentucky College of Medicine, Lexington, KY USA; ^2^ University of Kentucky, Lexington, KY USA; ^3^ University of Kentucky Sanders‐Brown Center on Aging, Lexington, KY USA

## Abstract

**Background:**

Past studies show that amygdala atrophy may be involved in the early stages of Alzheimer's disease (AD). AD is characterized by progressive cognitive decline, and changes in the amygdala—interconnected with various brain regions involved in cognition—could contribute to memory and emotional processing impairments. Specifically, changes in amygdala volumes may affect these neural networks, contributing to cognitive decline through disrupted communication between brain regions. The present study tests the association between amygdala volumes and non‐emotional memory retrieval and the sex differences in the association.

**Method:**

Participants (m_age_= 77.05, SD = 7.48; 25 females) from the University of Kentucky Alzheimer’s Disease Center cohort completed two trials of delayed match to sample task inside the 3T Siemens magnetic resonance imaging scanner.

**Result:**

We found that increased volume in the left hippocampus (p < .05, r = .31) and left amygdala (p < .05, r = .30) were associated with faster non‐match distractor response times (RTs) in the second trial. The result further shows significant sex differences whereby there was a significant negative association between the left hippocampus volume and RTs of non‐match distractors in the first trial (r = .40, p < .05) in females but not in males. Also, in females, the left amygdala volume had a significant positive association with accuracy (R2 = .40, p < .05) and a negative association with RTs of non‐match distractors for both trials (Trial 1: r = .43, p < .05; Trial 2: r = .47, p < .05).

**Conclusion:**

Investigating correlations between amygdala volumes and non‐emotional memory retrieval has implications for understanding cognitive processes, identifying markers of neurological disorders, addressing individual differences, and advancing both clinical and basic neuroscience research.

